# Clinical Study of Ultrasonographic Risk Factors for Central Lymph Node Metastasis of Papillary Thyroid Carcinoma

**DOI:** 10.3389/fendo.2021.791970

**Published:** 2021-11-30

**Authors:** Yang Guang, Wen He, Wei Zhang, Hongxia Zhang, Yukang Zhang, Fang Wan

**Affiliations:** Department of Ultrasound, Beijing Tian Tan Hospital, Capital Medical University, Beijing, China

**Keywords:** papillary thyroid carcinoma (PTC), contrast enhanced ultrasound (CEUS), ultrasonographic, superb microvascular imaging (SMI), central lymph node metastasis (CLNM)

## Abstract

**Background:**

Thyroid cancer is the most common malignancy of the endocrine system worldwide. Papillary thyroid cancer (PTC) is the most common pathologic type. The preoperative diagnosis of PTC and central lymph node metastasis (CLNM) or metastatic tendency is of great clinical significance to the diagnosis, treatment and prognosis of these patients. This study was conducted to investigate the correlation between ultrasound features and central CLNM of PTC.

**Methods:**

This study retrospectively analyzed patients who underwent PTC surgery and central lymph node dissection in the Department of Surgery, Beijing Tiantan Hospital, from January 2019 to February 2020. According to the inclusion and exclusion criteria, data from 136 patients were ultimately included, and the clinical and ultrasonic data of the patients were analyzed by multivariate regression to evaluate the correlation among grayscale ultrasound (US), superb microvascular imaging (SMI) and contrast-enhanced ultrasound (CEUS) features of thyroid nodules and CLNM of PTCs.

**Results:**

The multivariate analysis showed that tumor size, multifocality, microcalcification characteristics, SMI vascularization, and CEUS evaluation of contact with the adjacent capsule were correlated with PTC metastasis (*P*=0.008, *P*=0.001, *P*=0.028, *P*=0.041, and *P*< 0.001, respectively). Comparisons of the area under the ROC curves revealed that the area under the ROC curve of the degree of nodular invasion into the thyroid capsule was the largest (AUC: 0.754). The sensitivity and specificity for evaluating CLNM of PTC were 67.7% and 83.1%, respectively.

**Conclusions:**

Ultrasound characteristics of the following features are associated with a high risk of lymph node metastasis in PTCs: maximum diameter of nodules ≥1 cm, multifocality, ≥5 microcalcifications, abundant blood flow of SMI in nodules and nodule contact with the thyroid capsule ≥25% under CEUS. Ultrasound has clinical value in the preoperative evaluation of CLNM of PTCs.

## Background

Thyroid nodules have a high incidence among the global population, with an adult incidence of 50-60%, of which malignant tumors, namely, thyroid cancer, account for approximately 5% (1). In recent years, the incidence of thyroid cancer has been on the rise, making it the most common malignant tumor of the endocrine system in the world (2). Papillary thyroid cancer (PTC) is the most common pathologic type, accounting for 80 - 90% of thyroid cancer (3). Most cases of PTC have low malignancy, and it has been widely considered a disease with slow progression and good prognosis (4). However, approximately 30-80% of PTC patients develop lymph node metastasis, and some studies have shown that lymph node metastasis is associated with disease recurrence (5–7). When central lymph node metastasis (CLNM) is found in patients after surgery, a second operation is often necessary, increasing not only the difficulty of surgery but also the risk of complications such as recurrent laryngeal nerve injury and reduced parathyroid function; moreover, CLNM is an important risk factor for PTC recurrence and a reduced survival rate (8).

Therefore, preoperative evaluation of PTCs with or without CLNM is of great significance for the operation and prognosis of patients. At present, ultrasonography is an important imaging method for the diagnosis of PTCs, and superb microvascular imaging (SMI) and contrast-enhanced ultrasound (CEUS) techniques provide new methods for the differential diagnosis of thyroid cancer. SMI is an innovative ultrasound Doppler technique with several benefits: low-velocity flow visualization, high resolution of images, minimal motion artifacts, and high frame rates. SMI can also show detailed information about blood flow in the tumor. Previous reports have shown that CEUS could contribute to more accurate detection of external thyroidal extension (ETE) than grayscale ultrasound (US) (9, 10). Previous studies have reported that ultrasonic characteristics of PTC lesions have predictive value for the aggressiveness of PTCs (11–13). However, the relationship between multimodal US characteristics of PTCs and CLNM is still inconclusive and need further study. Therefore, in this study, the factors related to the ultrasonic characteristics of preoperative grayscale US combined with CEUS and SMI of PTCs were explored through multi-factor analysis to explore the predictive value for CLNM of PTCs, in order to provide a clinical basis for the early diagnosis.

## Methods

### Study Subjects

Clinical and ultrasonographic data of patients who underwent radical thyroid carcinoma and central lymph node dissection in the Department of General Surgery, Beijing Tiantan Hospital, Capital Medical University, from January 2019 to February 2020 were retrospectively collected. This study was approved by the Beijing Tian Tan Hospital Ethics Committee. A total of 136 patients with PTCs confirmed by postoperative pathology were included. The inclusion criteria were as follows: ① age between 20 and 80, male and female; ② At least one suspected malignant thyroid nodule (ACR TI-RADS 4 or TI-RADS 5) (14) was found by ultrasound examination within one month before surgery, which was clinically diagnosed as PTC and confirmed by postoperative pathology of thyroidectomy at our hospital (unilateral lobectomy and isthmus resection or total thyroidectomy with central lymph node dissection; if there are lateral neck metastases, multifunctional neck node preservation dissection should be performed); ③ patients undergoing thyroid surgery for the first time; ④ patients who have no contraindications to SonoVue, are in good mental state and can cooperate with the examination; ⑤ patients who agree to participate in the study. The exclusion criteria were as follows: ① PTC without regional lymph node dissection; ② two or more thyroidectomies; ③ other head and neck malignancies; and ④ a history of radiotherapy of the head and neck.

### Ultrasonography Examination and Imaging Analysis

US performed with Canon Aplio 500 or Aplio 900 sonographic scanners (Canon Medical Systems, Tokyo, Japan) that were equipped with 5-14 MHz or 4-18 MHz linear probes. CEUS imaging was performed using low mechanical index pulse reverse harmonic imaging technology. Sonovue (Bracco) was injected into the superficial elbow vein by mass injection, followed by 5 mL of normal saline.

Patients were placed in the supine or lateral decubitus position with the neck fully exposed and then observed by grayscale US and SMI. CEUS was performed on a section that could simultaneously show partial normal thyroid tissue and intact thyroid nodules. The patient was instructed to avoid swallowing and maintain calm and steady breathing after the injection of contrast agent. Dynamic contrast-enhanced images were continuously observed, and the relationship between nodules and adjacent thyroid capsules after CEUS was observed for a minimum of 2 min and recorded on the internal hard drive of the US system.

The grayscale US, SMI and CEUS images of all patients were analyzed by two sonographers, neither of whom performed any examinations in this study. The US and CEUS images were reviewed independently by two reviewers who specialized in thyroid disease diagnosis, each with more than 10 years of experience in thyroid US and CEUS. For any disagreements about the diagnosis, the final examination result was resolved by consensus or by the judgment of a third reviewer (with 15 years more experience in thyroid CEUS).

**Figure 1:**
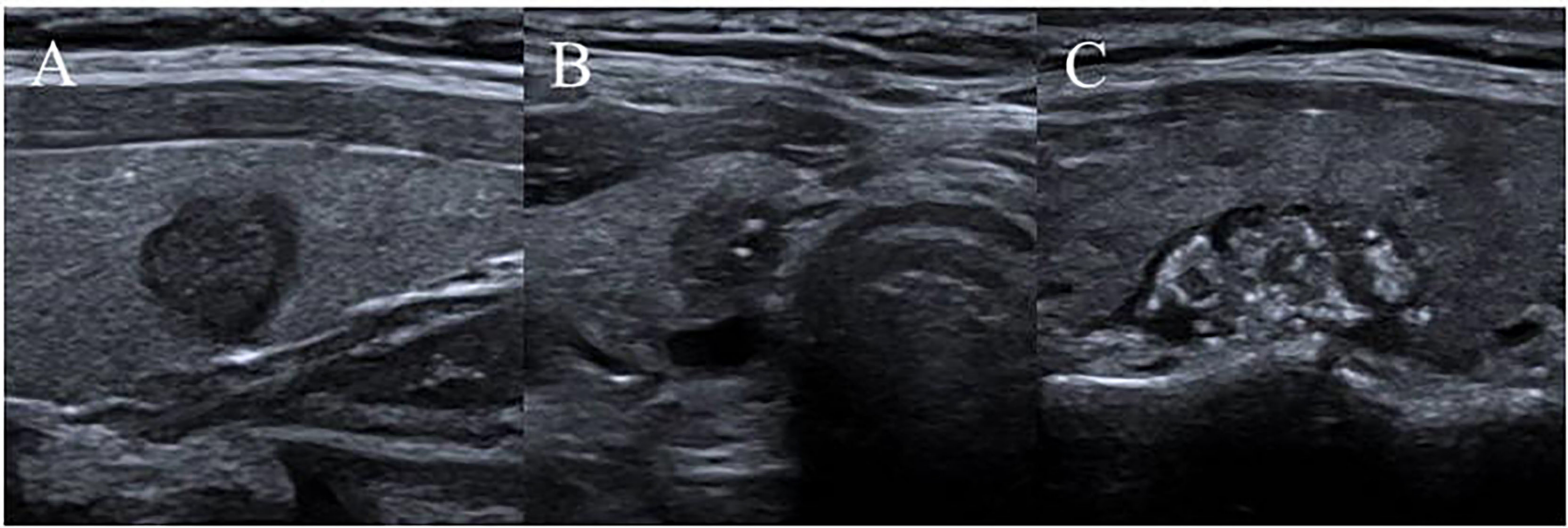
Microcalcifications classification of nodules. **(A)** no punctate echogenic foci; **(B)** less than 5 punctate echogenic foci; **(C)** more than 5 punctate echogenic foci.

**Figure 2:**
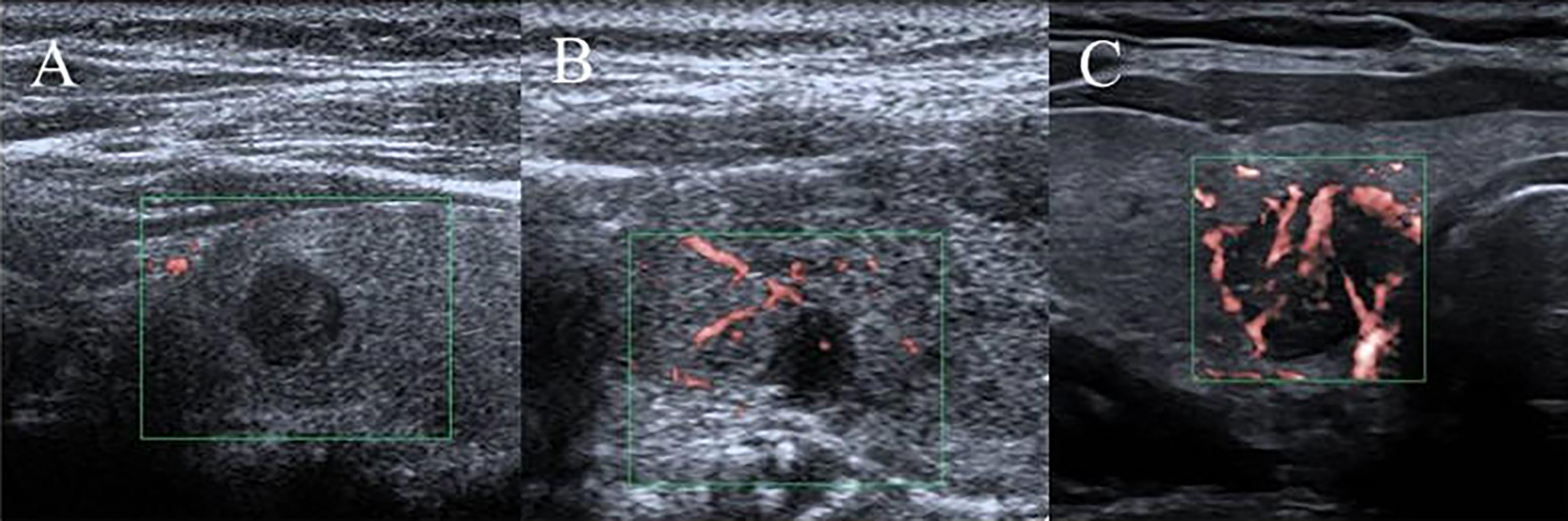
SMI vascularization classification of nodules. **(A)** Grade 0, no blood flow in the nodule; **(B)** Grade I, the nodules show a small amount of blood flow, only a few spots of blood flow or one long vessel penetrating into the nodule (more than half of the maximum diameter of the nodule); **(C)** Grade II, there is abundant blood flow inside the nodule, with 5 or more punctate blood flow or 2 long vessels.

**Figure 3:**
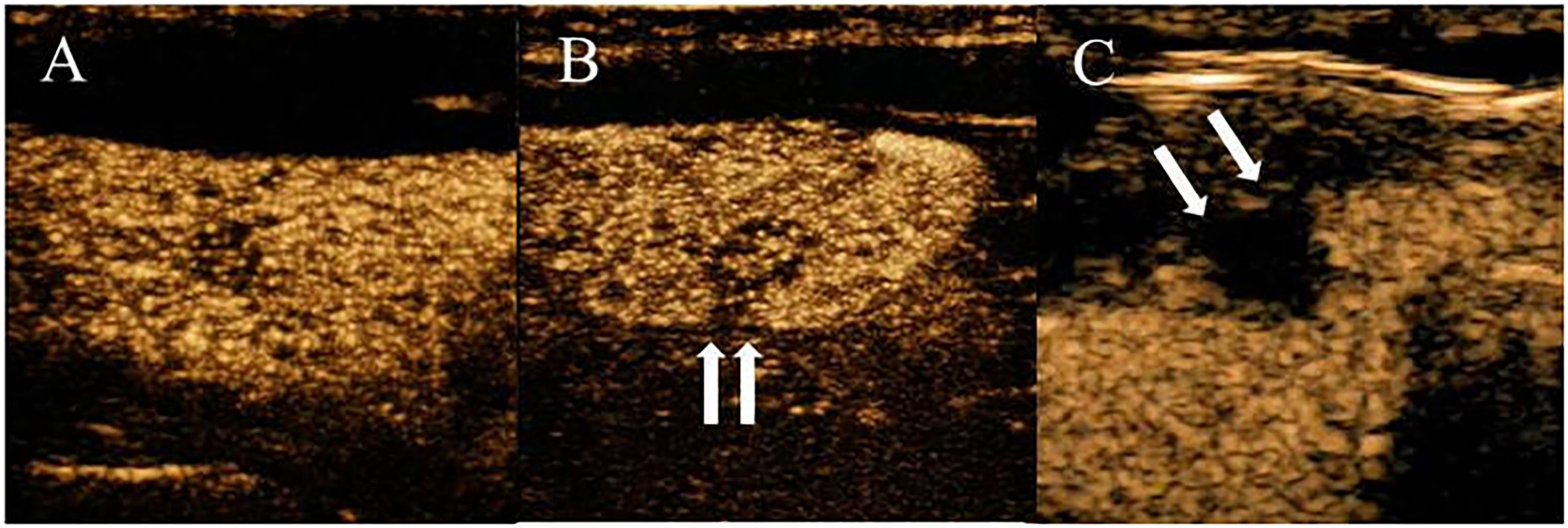
PTC with a variable degree of contact on CEUS imaging. **(A)** CEUS shows the nodule was not contact with the thyroid capsule. **(B)** CEUS shows the percentage of the nodule that contacted the thyroid capsule was less than 25% contact. **(C)** CEUS shows the percentage of the nodule that contacted the thyroid capsule was more than 25% contact.

Ultrasound characteristics of each nodule included as follows: nodule size (nodule maximum diameter < 1cm or ≥1cm);Multifocal PTC was defined as more than one lesion observed in a paraffin section, and the lesion with the maximum diameter or the most suspicious dominant nodule was considered for further analysis; Marked hypo-echogenicity (lower than the cervical band muscle); classification of margin was made as whether spiculated margin; taller- than-wide-shape (anteroposterior/transverse diameter ratio ≥ 1); The threshold value of microcalcifications was set as 1 mm, and microcalcifications were divided into two categories: no or less than 5 and more than 5 punctate echogenic foci, shown in **Figure 1**; SMI vascularization of nodules was classified according to the Adler standard (15) into the following 3 levels: Grade 0, no blood flow in the nodule; Grade I, the nodules show a small amount of blood flow, only a few spots of blood flow or one long vessel penetrating into the nodule (more than half of the maximum diameter of the nodule); and Grade II, there is abundant blood flow inside the nodule, with 5 or more punctate blood flow or 2 long vessels (shown in **Figure 2**); Contact of the thyroid mass with the thyroid capsule, namely, >25% contact with the adjacent capsule, is the most accurate measurement for predicting extrathyroidal extension (16). In this study, the relationship between nodules and adjacent thyroid capsules was evaluated after CEUS (the relationship between nodules and adjacent thyroid capsules was divided into two categories according to the contact area between nodules and capsules, < 25% and ≥25%, as shown in **Figure 3**). The above observations were evaluated using the longitudinal and transverse sections of multiple images. Intraobserver and interobserver agreements were evaluated (**Table 1**).

**Table 1:** Intraobserver and interobserver agreement on the ultrasonographic characteristics.

	Intraobserver agreement*	Interobserver agreement*
**Tumor size (cm)**	0.90 ± 0.02 (0.87-0.95)	0.82 ± 0.03 (0.75-0.89)
**Multifocality**	0.95 ± 0.02 (0.86-0.98)	0.89 ± 0.06 (0.84-0.94)
**Marked hypo-echogenicity**	0.76 ± 0.04 (0.73-0.89)	0.67 ± 0.16 (0.56-0.81)
**Spiculated margin**	0.87 ± 0.04 (0.83-0.02)	0.54 ± 0.06 (0.41-0.77)
**Taller than-wide shape**	0.87 ± 0.03 (0.80-0.91)	0.68 ± 0.07 (0.51-0.72)
**Microcalcification**	0.80± 0.03 (0.67-0.87)	0.67 ± 0.04 (0.59-0.75)
**SMI Vascularization**	0.78 ± 0.04 (0.69-0.83)	0.65 ± 0.03 (0.52-0.71)
**Contact with the adjacent capsule**	0.72 ± 0.03 (0.66-0.81)	0.67 ± 0.06 (0.56-0.78)

*Interobserver and intraobserver agreement was estimated using Cohen’s kappa or weighted kappa statistic.

Values were presented as kappa value ± standard error with the confidence interval.

### Statistical Analysis

We divided the patients into two groups, positive and negative, in accordance with the status of CLNM. The independent two-sample t-test was used to compare the differences in the mean of variables with a normal distribution between the two groups. Categorical variables are presented as percentages and compared with the chi-square test or Fisher’s exact test, as appropriate. All variables were included in the multivariate logistic model, and their corresponding odds ratios (ORs) and 95% confidence intervals (CIs) were calculated. A *p*-value <0.05 was considered statistically significant. All statistical analyses were performed using SPSS software version 22.0 (SPSS, Chicago, IL, USA).

## Results

### Baseline Characteristics of the Study Subjects

A total of 136 patients were included in this study. By the end of follow-up, all the patients were healthy and alive without distant metastasis or death caused by tumors. There were 65 cases with CLNM confirmed by postoperative pathology, the metastasis rate was 47.8%, and 71 cases did not exhibit CLNM. The baseline characteristics of the patients and US features of the thyroid nodules from the final histology analysis are presented in **Table 2**.

**Table 2:** Ultrasonographic characteristics of PTCs.

Characteristics		The status of metastatic lymph nodes		*P* Value
		Negative (n = 71)	Positive (n = 65)	
**Gender**				0.891
	Male (n, %)	20 (28.2%)	19 (29.2%)	
	Female (n, %)	51 (71.8%)	46 (70.8%)	
**Age (years)**				0.039
	<45 (n, %)	30 (42.3%)	39 (60.0%)	
	≥45 (n, %)	41 (57.7%)	26 (40.0%)	
**Tumor size (cm)**				<0.001
	<1 (n, %)	58 (81.7%)	30 (46.2%)	
	≥1 (n, %)	13 (18.3%)	35 (53.8%)	
**Multifocality**				0.002
	No (n, %)	57 (80.3%)	36 (55.4%)	
	Yes (n, %)	14 (19.7%)	29 (44.6%)	
**Marked hypo-echogenicity**				0.278
	No (n, %)	18 (25.4%)	22 (33.8%)	
	Yes (n, %)	53 (74.6%)	43 (66.2%)	
**Spiculated margin**				0.266
	No (n, %)	12 (16.9%)	16 (24.6%)	
	Yes (n, %)	59 (83.1%)	49 (75.4%)	
**Taller-than-wide shape**				0.277
	No (n, %)	19 (26.8%)	23 (35.4%)	
	Yes (n, %)	52 (73.2%)	42 (64.6%)	
**Microcalcification**				0.005
	<5 (n, %)	34 (47.9%)	16 (24.6%)	
	≥5 (n, %)	37 (52.1%)	49 (75.4%)	
**SMI Vascularization**				<0.001
	0-I (n, %)	44 (62.0%)	18 (27.7%)	
	II (n, %)	27 (38.0%)	47 (72.3%)	
**Contact with the adjacent capsule**				<0.001
	<25% (n, %)	59 (83.1%)	21 (32.3%)	
	≥25% (n, %)	12 (16.9%)	44 (67.7%)	

In the matched dataset, there were statistical differences in age, tumor size, multifocality, microcalcification, SMI vascularization and contact with the adjacent capsule on CEUS imaging between the lymph node metastasis-positive group and the lymph node metastasis-negative group (all *P <*0.05). There were no significant differences in terms of sex, marked hypoechogenicity, spiculated margin or taller-than-wide shape between the two groups (**Table 2**).

### Predictors of CLNM

To define the predictors of CLNM, we carried out multivariate logistic regression analysis using clinical and US features. In the multivariate analysis, nodule size (nodule maximum diameter ≥ 1 cm) (OR 4.437; *P*=0.008; 95% CI 1.484-13.266), number of nodules (Multifocality) (OR 7.106; *P*=0.001; 95% CI 2.280-22.150), microcalcification characteristics (≥5 microcalcifications) (OR 3.136; *P*=0.028; 95% CI 1.128-8.718), SMI vascularization characteristics (abundant blood flow of SMI, Grade II) (OR 2.780; *P*=0.041; 95% CI 1.043-7.410) and contact relationship with the capsule (nodule contact with the thyroid capsule ≥25% under CEUS) (OR 8.698; *P* < 0.001; 95% CI 3.051-24.798) were found to be independent risk factors for CLNM in PTC (all *P* < 0.05, **Table 3**).

**Table 3:** Multivariate analysis of ultrasonographic characteristics of CLNM from PTCs.

Characteristic	OR	95% CI	*P*-value
**Gender**	1.012	0.350-2.925	0.982
**Age (years)**	0.448	0.173-1.156	0.097
**Tumor size (cm)**	4.437	1.484-13.266	0.008
**Multifocality**	7.106	2.280-22.150	0.001
**Marked hypo-echogenicity**	0.654	0.217-1.971	0.451
**Spiculated margin**	0.890	0.259-3.057	0.853
**Taller-than-wide shape**	1.140	0.392-3.309	0.810
**Microcalcification**	3.136	1.128-8.718	0.028
**SMI Vascularization**	2.780	1.043-7.410	0.041
**Contact with the adjacent capsule**	8.698	3.051-24.798	<0.001

### Suspicious Preoperative Features in Predicting Lymph Node Metastasis

**Figure 4:**
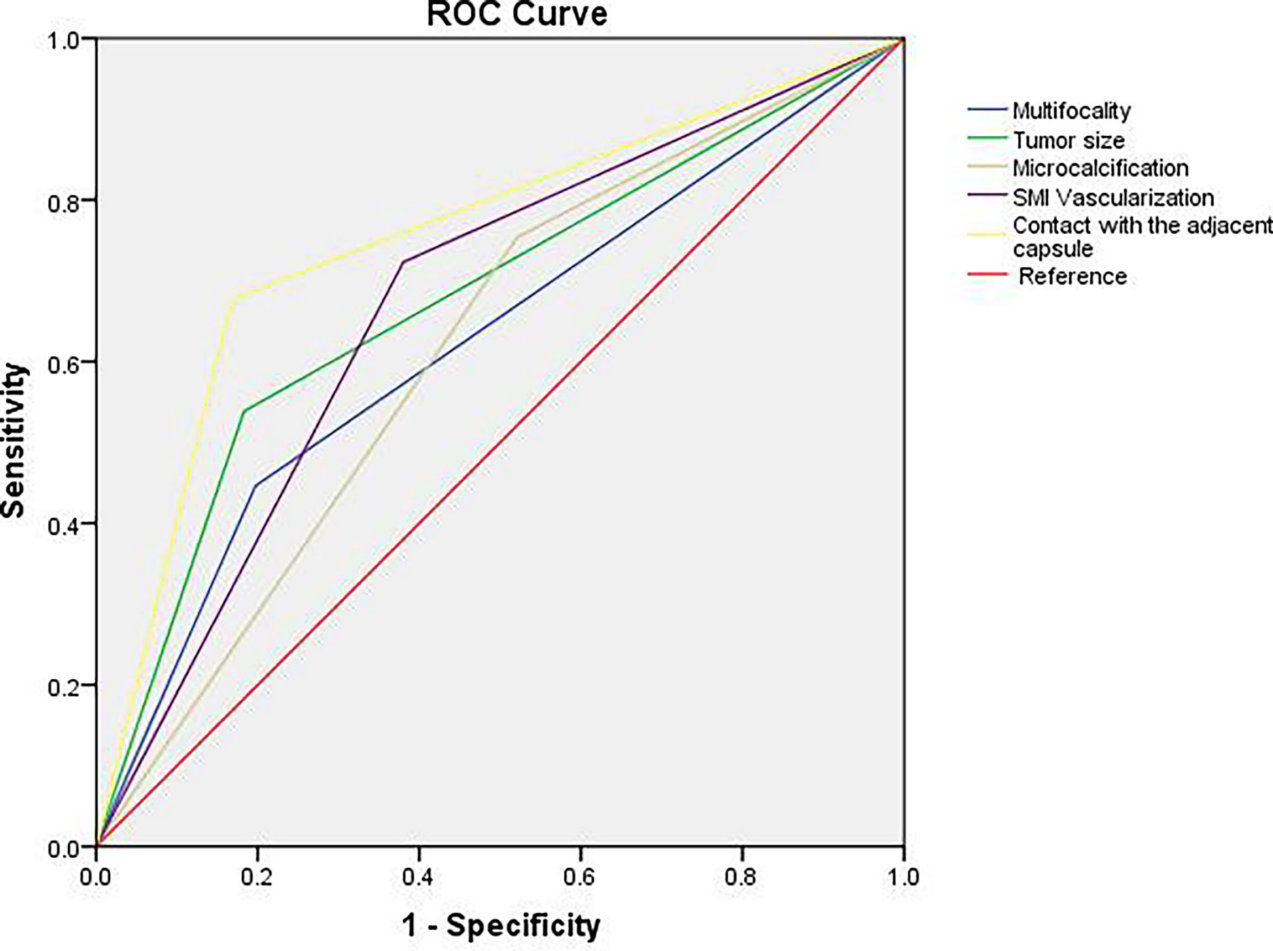
ROC curve analysis for predicting central lymph node metastasis. AUC for contact with the adjacent capsule was the largest (AUC: 0.754), the sensitivity was 67.7% and the specificity was 83.1%.

According to the receiver operator characteristic curve (ROC) curve analysis, the AUC for contact with the adjacent capsule was the largest (AUC: 0.754). Thus, we considered contact with the adjacent capsule ≥25% on CEUS as an indicator of a high risk of lymph node metastasis, with sensitivity and specificity values of 67.7% and 83.1%, respectively (**Table 4** and **Figure 4**).

**Table 4:** Predictive value analysis of CLNM of PTCs.

Independent predictors	AUC (95%CI)	Sensitivity (%)	Specificity (%)
**Tumor size (cm)**	0.678 (0.586-0.769)	53.8	81.7
**Multifocality**	0.624 (0.530-0.719)	44.6	80.3
**Microcalcification**	0.616 (0.522-0.711)	75.4	47.9
**SMI Vascularization**	0.671 (0.580-0.763)	72.3	62.0
**Contact with the adjacent capsule**	0.754 (0.670-0.838)	67.7	83.1

## Discussion

PTCs are the most common pathological type of thyroid cancer, with a 10-year survival rate of more than 90% (17). Previous studies have found that CLNM is the most common mode of PTC metastasis, and approximately 40-90% of PTC patients develop CLNM (18–21). At present, surgery is still the main treatment for PTCs, and one of the controversial aspects of PTC surgery is whether prophylactic central lymph node dissection is necessary. Accurate preoperative assessment of lymph node metastasis is helpful for designing a reasonable surgical plan and is the key to reducing the local recurrence rate and avoiding reoperation. Lymph node metastasis is therefore of great significance for clinical staging and postoperative recurrence rate assessments (22). In some patients with early metastasis and a lack of distinguishing features, the presence or absence of central lymph node helps to determine the surgical plan for PTCs and is closely related to the prognosis and survival of PTC patients. If potential metastatic lymph nodes are ignored, a second operation may be needed, which will be affected by the original operation scar and changes to the anatomical structure, increasing the difficulty of the operation and the incidence of complications such as phrenic nerve palsy, brachial plexus nerve palsy, cranial nerve injury, chylous leakage, parathyroid gland and recurrent laryngeal nerve injury (23). Therefore, the accurate preoperative assessment of CLNM is beneficial to the preoperative staging of PTCs. It also has important reference value for the selection of surgical methods and patient outcomes and provides a more accurate and objective basis for the individualized treatment decision of PTCs. Several studies have reported that ultrasonic characteristics of PTC lesions such as maximum diameter of the nodule, multifocality, microcalcification, and capsular invasion and patient characteristics such as sex and age have predictive value for the aggressiveness of PTCs; however, these findings are still inconclusive and need further study (11–13). Thus, in this study, 136 cases of PTC were retrospectively analyzed to explore the ultrasonic risk factors related to CLNM and to provide some reference for the selection of surgical procedures for PTC.

The size of the nodule is considered to be a major factor in postoperative pathological staging and prognostic risk grading of PTC patients. The size of the nodule is positively correlated with the growth rate and the degree of tissue infiltration and is prone to CLNM (24). In this study, multivariate regression analysis showed that the maximum diameter (≥1 cm) of the nodule was associated with CLNM, which was consistent with other reported findings (25–27). Multifocality is common in PTCs, with a reported incidence of approximately 18-87% (28, 29). Studies have shown that multifocality is associated with an increased risk of lymph node metastasis and recurrence and is considered a high risk factor for disease progression (30), which is consistent with the results of this study. In this study, multivariate analysis showed that multifocality was an independent risk factor for CLNM in PTCs. Previous studies have shown that microcalcification is due to calcium salt deposition caused by the proliferation of blood vessels and fibrous tissue, and microcalcification reflects the characteristics of rapid growth in cancer tissues (31). Bai Y et al. (32, 33) retrospectively analyzed the pathological characteristics of PTC patients to explore the clinical significance of different pathological types of calcification in PTCs. Patients with sandy body and interstitial calcification were found to have higher cancer stages and more lymph node metastases. In this study, multivariate analysis showed that ≥5 microcalcifications was an independent risk factor for CLNM in PTCs. Therefore, if the maximum tumor diameter is ≥1 cm and multifocality or multiple microcalcifications are found in the nodules, further careful evaluation should be performed for CLNM.

Previous studies have shown that with the growth of thyroid cancer, the vessels in the tumor are induced by angiogenic factors such as vascular endothelial growth factor (VEGF) and form a disordered vascular network (34). Studies have reported that the expression of VEGFR1 is an important predictor of lymph node metastasis, with one showing that NRP2 plays an important role in the regulation of VEGF-induced invasion and migration *in vitro* (35). With the increase in tumor angiogenesis, the contact between the active cells at the tumor margin and lymphatic vessels increases, making the tumor prone to metastasize into the lymphatic system. SMI imaging technology uses intelligent calculation and measurement methods to effectively distinguish between low-velocity blood flow and Doppler signals generated by tissue movement. The low-speed blood flow information filtered out by traditional Doppler ultrasound can be extracted and displayed to retain even the most subtle low-speed blood flow information and truly reflect the blood perfusion situation of the nodular tissue. Therefore, in this study, the SMI technique was used to evaluate the characteristics of blood flow in the nodule. The results showed that the characteristics of SMI in PTC nodules (Grade II) were independent influencing factors for CLNM in PTCs, suggesting that the degree of rich and disorderly blood flow in PTC nodules could predict the possibility of CLNM.

ETE refers to thyroid cancer that extends beyond the thyroid capsule and extends to tissues outside the thyroid gland. It is an important invasive feature of PTCs, and the incidence of ETE has been reported to be 5-45% (36, 37). The invasion of nodules into the capsule will lead to changes in the morphology of the capsule, resulting in the disruption of the continuity of the capsule, invasion and breakthrough of the capsule, and metastasis to adjacent tissues and distant lymph nodes. In this study, we found that observation of the anterior thyroid capsule by grayscale US is affected by ultrasound near-field artifacts, while observation of the lateral and posterior thyroid capsules is affected by blood vessels and the trachea, which cannot be clearly shown. Therefore, we observed the relationship between nodules and thyroid capsule by CEUS, which could more clearly illustrate the relationship between nodules and thyroid capsule. Previous studies (16) have indicated that contact with an adjacent thyroid capsule > 25% is the most accurate measurement for predicting extrathyroidal extension. Based on this criterion, we subdivided PTCs based on contact with the adjacent capsule using a >25% threshold under CEUS. The results suggested that nodule contact with the thyroid capsule ≥25% was an independent risk factor for CLNM of PTCs. The mechanism may be related to the fact that PTCs are lymphophilic tumors, and the thyroid gland has a rich lymphatic network. Once the tumor breaks through the capsule, it could easily cause lymph node metastasis in the central region (38).

Comparisons of the area under the ROC curves showed that the assessment of contact with the adjacent capsule under CEUS was an independent risk factor for CLNM, with the largest area under the curve and the highest specificity (83.1%) in the assessment of CLNM of PTCs. Microcalcification (≥5) had the highest sensitivity (75.4%) as an independent risk factor for evaluating CLNM in PTCs.

However, there are still some limitations to this study. First, this was a retrospective study with a relatively insufficient sample size. Second, more ultrasonic features, such as CEUS and elastography, still need to be included to evaluate their correlation with PTC lymph node metastasis. Therefore, additional prospective studies are needed to obtain more objective evidence for clinical use, which is also a further research direction.

## Conclusion

In this study, grayscale US, SMI and CEUS characteristics of PTC were analyzed. The following features are associated with a high risk of lymph node metastasis in PTCs: maximum diameter of nodules ≥1 cm, multifocality, ≥5 microcalcifications, abundant blood flow of SMI in nodules and nodule contact with the thyroid capsule ≥25% under CEUS. These features might help provide more tailored management strategies for patients with suspicious thyroid nodules.

## Data Availability Statement

The raw data supporting the conclusions of this article will be made available by the authors, without undue reservation.

## Ethics Statement

The studies involving human participants were reviewed and approved by Beijing Tian Tan Hospital Ethics Committee. The patients/participants provided their written informed consent to participate in this study.

## Author Contributions

YG and WH—conceived the idea for the work. WZ and HZ—designed the analysis. YG and YZ—analyzed the data. YG—wrote the manuscript. YG and FW—interpreted the data and reviewed the manuscript. WH—provided overall supervision for the work. All authors contributed to the article and approved the submitted version.

## Funding

National Natural Science Foundation of China (grant no. 81901744); Natural Science Foundation of Beijing (grant no. 7204255).

## Conflict of Interest

The authors declare that the research was conducted in the absence of any commercial or financial relationships that could be construed as a potential conflict of interest.

## Publisher’s Note

All claims expressed in this article are solely those of the authors and do not necessarily represent those of their affiliated organizations, or those of the publisher, the editors and the reviewers. Any product that may be evaluated in this article, or claim that may be made by its manufacturer, is not guaranteed or endorsed by the publisher.

## Abbreviations

PTC, Papillary thyroid cancer; SMI, Superb microvascular imaging; CEUS, Contrast-enhanced ultrasound; ROC, Receiver operating characteristic; AUC, Area under the curve; ETE, External thyroidal extension; US, Ultrasound.
